# His‐163 is a stereospecific proton donor in the mechanism of d‐glucosaminate‐6‐phosphate ammonia‐lyase

**DOI:** 10.1002/1873-3468.14469

**Published:** 2022-08-29

**Authors:** Robert S. Phillips, Kaitlin L. Anderson, Declan Gresham

**Affiliations:** ^1^ Department of Chemistry University of Georgia Athens GA USA; ^2^ Department of Biochemistry and Molecular Biology University of Georgia Athens GA USA; ^3^ Department of Genetics University of Georgia Athens GA USA; ^4^ Department of Cellular Biology University of Georgia Athens GA USA

**Keywords:** aminoacrylate intermediate, elimination reaction, pyridoxal‐5′‐phosphate, stereochemistry

## Abstract

d‐Glucosaminate‐6‐phosphate ammonia‐lyase (DGL) catalyzes the conversion of d‐glucosaminate‐6‐phosphate to 2‐keto‐3‐deoxyglutarate‐6‐phosphate, with stereospecific protonation of C‐3 of the product. The crystal structure of DGL showed that His‐163 could serve as the proton donor. H163A mutant DGL is fully active in the steady‐state reaction, and the pre‐steady‐state kinetics are very similar to those of wild‐type DGL. However, H163A DGL accumulates a transient intermediate with λ_max_ at 293 nm during the reaction that is not seen with wild‐type DGL. Furthermore, NMR analysis of the reaction of H163A DGL in D_2_O shows that the product is a mixture of deuterated diastereomers at C‐3. These results establish that His‐163 is the proton donor in the reaction mechanism of DGL.

## Graphical abstract

His‐163 is the stereospecific source of the proton on C‐3 of 2‐keto‐3‐deoxy‐d‐gluconate‐6‐phosphate produced from d‐glucosaminate‐6‐phosphate by d‐glucosaminate‐6‐phosphate ammonia‐lyase. This protonation step is not rate determining but may be necessary to avoid the release of a potentially cytotoxic intermediate.

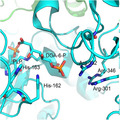

## Abbreviations

**KDG‐6‐P**, 2‐keto‐3‐deoxyglutarate‐6‐phosphate

**DGL, (4.3.1.29)**, d‐glucosaminate‐6‐phosphate ammonia‐lyase

**DGA‐6‐P**, d‐glucosaminate‐6‐phosphate

**DGA**, d‐glucosaminic acid

**PLP**, Pyridoxal‐5′‐phosphate

**Fig. 1:**
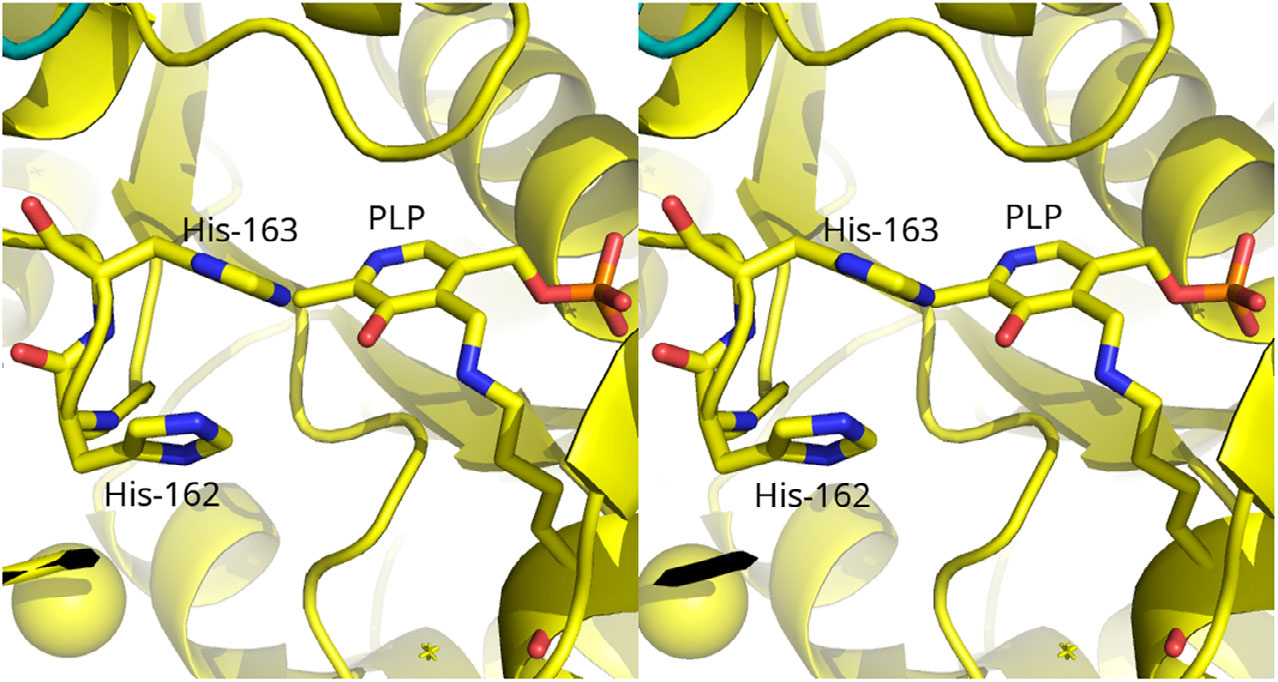
Crossed‐eye stereo view of the structure of DGL (PDB 7CLE), showing the proximity of His‐163 to the PLP. This figure was prepared with Pymol 2.3.0 (PyMOL Molecular Graphics System, Version 2.0 Schrödinger, LLC, New York, NY, USA).

**SVD**, singular value decomposition


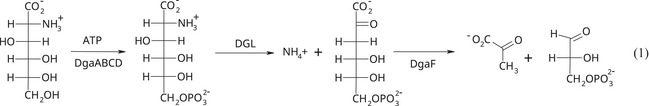
 *Salmonella enterica* serovar typhimurium and other related enterobacteria can utilize d‐glucosaminic acid (DGA, EC 4.3.1.29) as a sole carbon and nitrogen source through a pathway that we have recently described (Eq. 1) [1]. Uptake of DGA into cells occurs by a PTS transport system made up of DgaABCD that gives d‐glucosaminate‐6‐phosphate (DGA‐6‐P). The key step in this pathway is the deamination of DGA‐6‐P by d‐glucosaminate‐6‐phosphate ammonia‐lyase (DGL) to give 2‐keto‐3‐deoxygluconate‐6‐phosphate (KDG‐6‐P). Subsequently, an aldolase, produced by the DgaF gene, breaks down KDG‐6‐P to pyruvate and d‐glyceraldehyde‐3‐phosphate to enter the Entner–Doudoroff alternative glycolytic pathway. We previously created a homology model of DGL that predicted His‐162 or His‐163 would be possible catalytic groups, based on their proximity to the active site [2]. More recently, we have determined the X‐ray crystal structure of DGL, and we confirmed that His‐162 and His‐163 are located near the active site (Fig. 1) [3]. These histidine residues are strictly conserved in the sequences of DGL, even with relatively low sequence identity (Fig. S1). We found previously that the reaction of DGL in D_2_O gives a single diastereomeric 3‐deutero‐KDG‐6‐P, where the pro‐*R* proton of KDG‐6‐P is stereospecifically replaced by deuterium, resulting in an unusual inversion of stereochemistry in a β‐elimination reaction [3]. Thus, the unsaturated enamine intermediate must be protonated on the opposite face than water is eliminated from, possibly by conserved His‐163 as a proton donor. In the present study, we have mutated His‐163 to alanine to determine its function in the catalytic mechanism of DGL. The results are consistent with our prediction that His‐163 is the source of the proton on C‐3 of the KDG‐6‐P product.

## Materials and methods

### Materials

DGA‐6‐P was prepared from d‐glucosamine hydrochloride (Sigma‐Aldrich, St. Louis, MO, USA) as described previously [2]. Buffers and reagents were obtained from various commercial sources.

### Enzymes

Wild‐type DGL was prepared as described previously [1, 2]. H163A DGL was prepared by PCR mutagenesis with partially overlapping primers, as described by Liu and Naismith [4], with the wild‐type DGL plasmid as the template. The mutagenic primers are shown below with the mutagenic

Forward: 5′‐GCGCTACTGTATGTGAAATCACAC**GC**TTGCGTACAGAAAGGCATGTTGAGC‐3′

Reverse: 5′‐GCGCTACTGTATGTGAAATCACACGCTTGCGTACAGAAAGGCATGTTGAGC‐3′ bases indicated in bold. The PCR reaction used Phusion DNA polymerase (Thermo Fisher, Waltham, MA, USA), with annealing for 30 s at 55 °C and extension for 3 min at 72 °C for 30 cycles. The PCR product was then used to transform competent GC‐10 *E. coli* cells (Genesee Scientific). Single colonies were grown for plasmid purification to confirm the presence of the mutation by sequencing. The mutant plasmid was then used to transform competent *E. coli* BL21(DE3) (New England Biolabs, Ipswitch, MA, USA) for expression. An overnight culture in 5 mL LB with 100 μg·mL^−1^ ampicillin was used to innoculate 1 L of Studier ZYP‐5052 autoinduction medium [5] in a 2 L Erlenmeyer flask. The cells were grown for 24 h at 37 °C with shaking at 250 r.p.m. The cells were harvested by centrifugation for 15 min, then the cell pellet was resuspended in 30 mL 0.05 m potassium phosphate, pH 7, 0.3 m NaCl, and 0.1 mm PLP. The cell‐free extract was obtained by sonication for 4‐min in 1‐min intervals, with cooling on ice, followed by centrifugation for 90 min. The supernatant was applied to a Ni‐NTA Sepharose (GE Healthcare, Smyrna, GA, USA) affinity column equilibrated with 0.05 m potassium phosphate, pH 7, 0.3 m NaCl, 0.1 mm PLP, and washed with the buffer containing 20 mm imidazole until the 280 nm absorbance returned to baseline. The protein was eluted with the buffer containing 200 mm imidazole and concentrated in a centrifugal concentrator (Pall), then diluted with buffer without imidazole and concentrated. After concentration, the protein was frozen in aliquots at −80 °C.

### DGL assay

DGL was assayed as described previously [1, 2]. The assay mixture contained 0.1 m triethanolamine hydrochloride, pH 8, 0.2 mm NADH, 50 μm PLP, 16 μg·mL^−1^ rabbit muscle lactate dehydrogenase, 4 μL of crude DgaF, and various amounts of DGA‐6‐P. The reactions were followed on a Cary 100 UV–visible spectrophotometer at 340 nm and 37 °C. There was often a short lag (1–2 min) before the steady‐state rate was achieved. The rate was measured in the steady state, using Δε = 6.2 × 10^3^·m ^−1^·s^−1^ for the molar extinction coefficient of NADH at 340 nm. The data were fit to Eq. 2, the Michaelis–Menten equation [6], using the HYPERO program of Cleland [7]. Errors are given as standard errors based on the fits. The subunit molecular mass of both wild‐type and H163A DGL was assumed to be 39.3 kDa.

(2) $$V=V_{\max}\times \left[S\right]/\left(K_{m}+\left[S\right]\right)$$

### Stopped‐flow kinetics

The rapid‐scanning stopped‐flow kinetics were performed in an OLIS RSM‐1000 instrument equipped with a stopped‐flow mixer. Absorbance scans were collected from 250 to 800 nm at 1000 Hz for 1–2 s, or at 62 Hz for 60 s or longer. Solutions of wild‐type or H163A DGL (2 mg·mL^−1^) were mixed 1:1 with 20 mm ligand solutions in 0.1 m Tris–HCl, pH 8, at ambient temperature (~ 20 °C). The data were analyzed with the Global Works program provided by OLIS [8].

### Mass spectrometry

The sample contained 0.5 mg DGA‐6‐P, 1 mg NH_4_HCO_3_, 1 μL H163A DGL (35 μg), and 99 μL water. It was incubated for 30 min at room temperature, then analyzed by ESI‐MS in negative‐ion mode on a Bruker Impact II Q‐TOF instrument.

### NMR spectra

The samples for NMR contained 2.75 mg of DGA‐6‐P and 1.4 μL of 14.2 m NaOD to bring the pH up to 7 and 10 μL wild‐type or H163A DGL, in 600 μL D_2_O. After 30 min at room temperature, the spectra were collected on a Bruker Avance instrument at 400.14 MHz with presaturation for water suppression.

### Manual docking of DGA‐6‐P into DGL

The model of DGA‐6‐P in an extended conformation was prepared with ChemBio3D (Perkin Elmer, Inc., Billerica, MA, USA) and docked into the structure of DGL (PDB 7LCE) manually with COOT [9]. The phosphate group was placed near the cluster of Arg‐301, 332, and 346, and the amino group was placed in a position to form a bond to C4’ of the internal aldimine of PLP.

## Results and Discussion

### Steady‐state kinetic activity of H163A DGL

H163A DGL was prepared by PCR mutagenesis, expressed, and purified by Ni‐MAC, as previously described for wild‐type DGL [2, 3]. Although we had speculated that His‐162 and His‐163 might be responsible for the binding of the unmodified enzyme to the Ni‐MAC column, we found that the H163A mutant enzyme behaved similarly on the column as wild‐type DGL. The mutant enzyme has high steady‐state activity in the standard coupled assay with DgaF and LDH, very similar to that of wild‐type DGL (Table 1). A high‐resolution ESI‐MS of the reaction mixture of H163A DGL and DGA‐6‐P showed a major peak for (m ^−1^)^−^ at m/z = 275.00679 (predicted for C_6_H_10_PO_9_, 275.00570) (Fig. S2), showing that the reaction product of the mutant enzyme is also KDG‐6‐P.

**Table 1:** Steady‐state kinetic activity of wild‐type and H163A DGL.

	DGL	H163A DGL
*k*_cat_, s^−1^	27.4 ± 1.4^a^	20 ± 0.8
*k*_cat_/*K*_m_, m ^−1^·s^−1^	(1.97 ± 0.31) × 10^5^ ^a^	(6.0 ± 0.9) × 10^4^

^a^ From reference [3].

### Stopped‐flow kinetics of H163A DGL

**Fig. 2:**
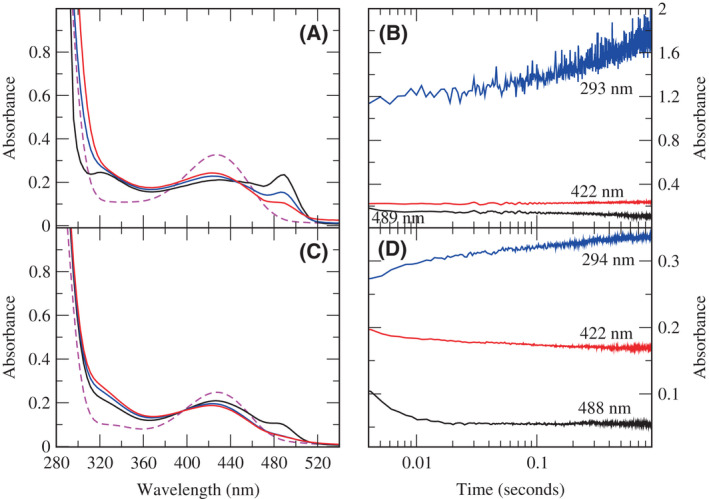
Rapid‐scanning stopped‐flow spectroscopy of H163A and wild‐type DGL with DGA‐6‐P in the early part of the reaction. (A) Reaction of H163A DGL. The enzyme spectrum is shown with the dashed magenta line. The first spectrum after mixing (0.001 s) is shown in black. The second spectrum is at 0.04 s (blue). The third spectrum is at 1.2 s (red). (B) Time courses for the reaction of H163A DGL in the early part of the reaction. Black, 489 nm; red, 422 nm; blue, 293 nm. (C) Reaction of wild‐type DGL. The enzyme spectrum is shown with the dashed magenta line. The first spectrum after mixing (0.001 s) is shown in black. The second spectrum is at 0.04 s (blue). The third spectrum is at 0.9 s (red). (D) Time courses for the reaction of wild‐type DGL in the early part of the reaction. Black, 488 nm; red, 422 nm; blue, 294 nm.

Wild‐type and H163A DGL were mixed with DGA‐6‐P in the rapid‐scanning stopped‐flow spectrophotometer. A peak at about 486 nm was seen in the first scan (ca. 2 m), and this peak decayed with a *k*_obs_ = 318 ± 30 s^−1^, along with an increase in absorbance in the 300–360 nm region (Fig. 2A,B). This is very similar to what is observed with wild‐type DGL, *k*_obs_ = 254 ± 40 s^−1^ (Fig. 2C), although the 486 nm peak is more prominent for the mutant enzyme. However, subsequently, there is a large increase in absorbance below 300 nm (Fig. 2B) with H163A DGL that is not observed with wild‐type DGL (Fig. 2D).

**Fig. 3:**
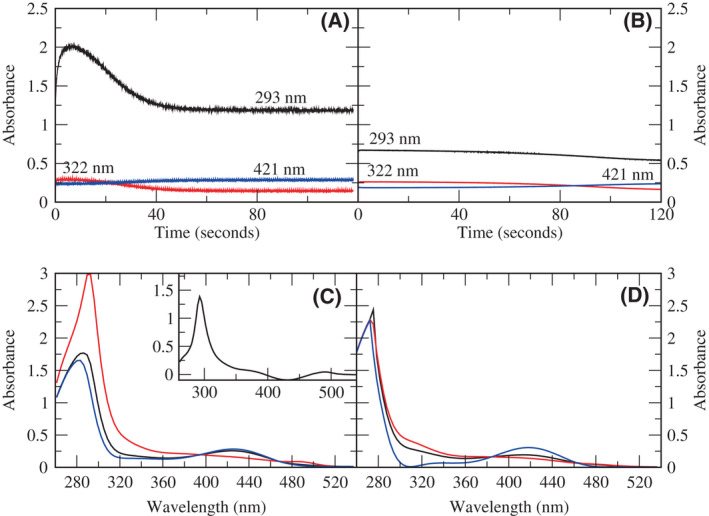
Time courses and SVD spectra for the reaction of H163A and wild‐type DGL with DGA‐6‐P. (A) Time courses for H163A DGL. Black, 293 nm; red, 322 nm; blue, 421 nm. (B) Time courses for wild‐type DGL. Black, 293 nm; red, 322 nm; blue, 421 nm. (C) SVD spectra for reaction of H163A DGL. Black, initial spectrum; red, intermediate spectrum; blue, final spectrum. The inset shows the difference between the black and red SVD spectra. (D) SVD spectra for reaction of wild‐type DGL. Black, initial spectrum; red, intermediate spectrum; blue, final spectrum.

The SVD spectra and time courses for the overall reaction of H163A DGL (Fig. 3A,C) show that the 293 nm absorbance reaches a maximum at 6 s and decays completely by 60 s, at which time the substrate has been consumed. The singular value decomposition (SVD) spectra for the reaction show an intermediate with a strong peak at 293 nm (Fig. 3C). The difference spectrum of SVD spectra 1 (black) and 2 (red) shows that the absorbance of this intermediate is about 1.4 (Fig. 3C, inset). The high absorbance of this intermediate suggests that it is not enzyme bound but has been released into the solution. Furthermore, the substrate concentration is 10 mm, while the enzyme concentration is 25 μm, so if it were enzyme bound, the intermediate would have a molar extinction coefficient of ≥56 000 m ^−1^·cm^−1^. No PLP enzyme intermediate is known, which has an absorbance at 293 nm with such a high molar extinction coefficient. We suggest that this transient intermediate is formed in millimolar concentrations and is the substituted aminoacrylate product of the β‐elimination of water from the 3‐position from DGA‐6‐P. The spectra and time courses for the overall reaction of wild‐type DGL (Fig. 3B) do not show the formation of a strongly absorbing intermediate in the UV region. Furthermore, the SVD spectra do not show any peak other than the protein peak at 280 nm (Fig. 3D).

### Stereochemistry of H163A DGL

**Fig. 4:**
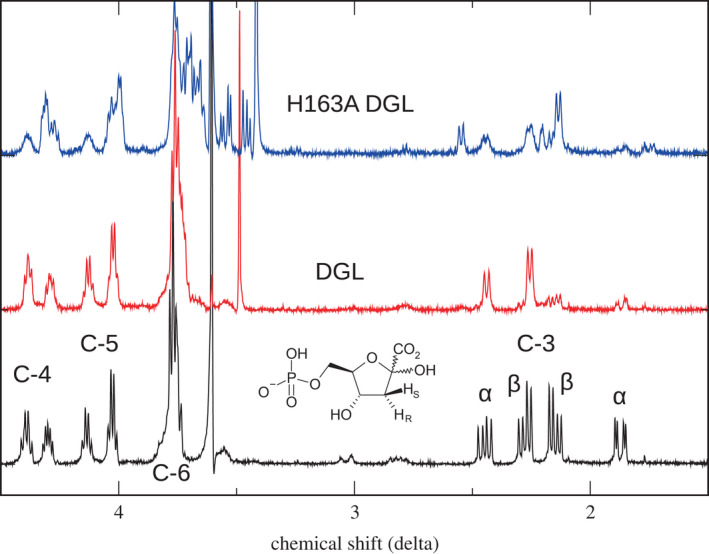
^1^H‐NMR spectra of KDG‐6‐P from reaction of wild‐type and H163A DGL. Black, spectrum in D_2_O of KDG‐6‐P from reaction of wild‐type DGL with DGA‐6‐P in H_2_O. Red, spectrum of KDG‐6‐P from reaction of wild‐type DGL with DGA‐6‐P in D_2_O. Blue, spectrum of KDG‐6‐P from reaction of H163A DGL with DGA‐6‐P in D_2_O. The two sets of peaks arise from the α‐ and β‐anomers.

The reaction of wild‐type and H163A DGL with DGA‐6‐P was performed in D_2_O, and the ^1^H‐NMR spectra were obtained (Fig. 4). Previously, we found by NMR that the protonation of the product is stereospecific, producing (3*R)*‐3‐deutero‐KDG‐6‐P together with trace amounts of the epimeric (3*S*)‐deuterated product and protiated KDG‐6‐P (Fig. 4, red spectrum) [3]. By contrast, the product of the reaction of DGA‐6‐P with H163A DGL in D_2_O shows that the product is an approximately equal mixture of the deuterated diastereomers (Fig. 4, blue spectrum), along with traces of protiated KDG‐6‐P. For reference, the spectrum of the KDG‐6‐P was obtained by the reaction with wild‐type DGL in H_2_O is shown (Fig. 4, black spectrum). The upfield peaks between 1.5 and 2.5 ppm arise from the diastereotopic methylene protons on C‐3, with the geminal coupling and vicinal coupling with the C‐4 H. The loss of the geminal coupling in the monodeuterated product results in doublets for the H's on C‐3. The spectrum is further complicated by the mixture of α‐ and β‐anomers and a small amount of the open chain. The mixture of deuterated isomers in the H163A DGL product can be seen not only for the resonances of the C‐3 hydrogens but also as multiple peaks for the upfield H's of C‐4, C‐5, and C‐6. Thus, removing the imidazole side chain of His‐163 has eliminated the stereospecific protonation of the KDG‐6‐P reaction product seen with wild‐type DGL. This likely occurs because the protonation of the enamine intermediate, released from the mutant enzyme, is now nonenzymatic and occurs in the solution after product release from the enzyme. These results provide support for our previous proposal, based on the X‐ray crystal structure, that His‐163 is a proton donor in the reaction mechanism [3].

### Reaction mechanism of H163A DGL

**Scheme 1:**
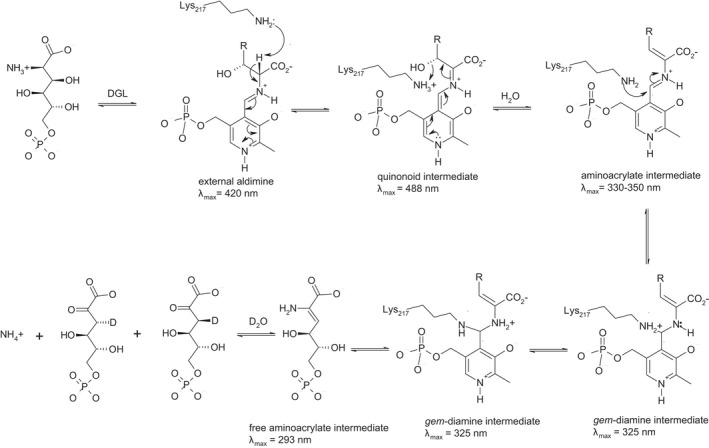
Mechanism of H163A DGL.

The proposed reaction mechanism for H163A DGL is shown in Scheme 1. The binding of the substrate for both wild‐type and H163A mutant DGL occurs within 2 ms to give the quinonoid intermediate with absorbance at about 488 nm. This intermediate decays by the elimination of the C‐3 OH, assisted by the Lys‐217 ε‐ammonium as a proton donor, to give a substituted aminoacrylate intermediate, with a rate constant of about 300 s^−1^, comparable to that of wild‐type DGL. If His‐163 is present, this aminoacrylate intermediate is subsequently protonated at C‐3 from the opposite face. However, this protonation must not be rate determining since the steady‐state kinetics are hardly affected by the mutation (Table 1). Subsequent to protonation, or in the absence of the proton donor, the next step is nucleophilic attack by the ε‐amino group of Lys‐217 on the aldimine bond of the aminoacrylate intermediate, giving a *gem*‐diamine. Given that quinonoid intermediate formation, water elimination, and aminoacrylate protonation steps are not rate limiting in the steady state, the rate‐limiting step must be product release. After proton transfer from Lys‐217 to the product nitrogen, the H163A mutant enzyme releases the enamine form of the product, whereas the wild‐type enzyme releases the imino form of the product from the *gem*‐diamine.

### Role of His‐162 in DGL

**Fig. 5:**
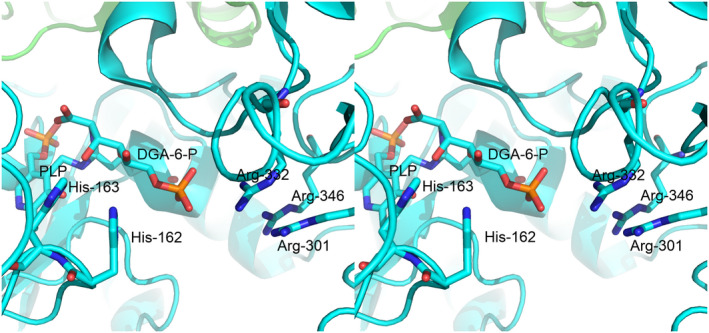
Crossed‐eye stereo structure of DGA‐6‐P docked manually into the active site of DGL (PDB 7LCE) to give a *gem*‐diamine.

His‐162 is a conserved residue in all sequences of DGL (Fig. S1). What is the function of His‐162 in DGL? A model was created *in silico* by manual docking of DGA‐6‐P, forming a *gem*‐diamine with the PLP (Fig. 5). The phosphate group is located near a cluster of 3 arginine residues, 301, 332, and 346, and the α‐carboxylate is located near Arg‐226. In this model, NE2 of His is about 4 Å from C‐3 of the substrate, the site of protonation. The NE2 of His‐162 is about 6 Å away from C‐3, but only about 4 Å from the O6, the bridging oxygen of the phosphate ester, of the substrate (Fig. 5). Thus, His‐163 is better positioned for proton transfer to the reaction intermediate than His‐162. Note that these histidines are located on a flexible loop. It is possible that the role of His‐162 is to anchor the loop by hydrogen bonding to O6 or to phosphate oxygen, thus positioning His‐163 for the proton transfer to C‐3 of the substrate.

### Physiological function of His‐163 in DGL

His‐163 is strictly conserved in all sequences of DGL, even those with relatively low (~ 40%) overall sequence identity (Fig. S1). Since the protonation of C‐3 of the product is not rate limiting (Table 1), why does the enzyme have a strictly conserved histidine residue to perform stereospecific protonation of the product? Enamine intermediates formed by elimination reactions of PLP‐dependent enzymes are known to be toxic to cells because they react as nucleophiles with the internal aldimines of many PLP‐dependent enzymes [10]. These enzymes are irreversibly inactivated by covalent bond formation [11, 12, 13]. Indeed, this has formed the basis for the design of irreversible inactivators of PLP‐dependent enzymes as potential drugs [14]. The formation of free aminoacrylates by PLP‐dependent enzymes is well‐documented, for example, in the reaction of threonine deaminase [10, 15]. PLP enzymes that catalyze β‐eliminations preferentially release iminopyruvate rather than 2‐aminoacrylate to avoid cytotoxicity [16]. There are enzymes, such as RidA, whose function is to break down these enamines, like 2‐aminoacrylate, even though the half life of these intermediates in aqueous solution at neutral pH is generally on the order of a few seconds [17]. If RidA enzymes are deleted from microbial genomes, the mutant cells have low viability [10]. Thus, if the enamine derived from KDG‐6‐P is also cytotoxic, there is obvious selection pressure to maintain the enzyme in a form that releases the nontoxic imino product rather than the potentially cytotoxic enamine product, even without direct effects on catalysis. Interestingly, there is no evidence that H163A DGL itself is undergoing inactivation by the enamine during turnover since the NMR and stopped‐flow studies indicated stoichiometric turnover. The stereospecificity of KDG‐6‐P protonation results from the spatial relationship of the His‐163 proton donor on the opposite face of the enzyme–substrate complex from the Lys‐217 in the active site.

## Conclusions

His‐163 is the stereospecific source of the proton on C‐3 of KDG‐6‐P produced from DGA‐6‐P by DGL. This protonation step is not rate determining but may be necessary to avoid the release of a potentially cytotoxic intermediate.

## Author contributions

KLA prepared the wild‐type enzyme and created a homology model of DGL. DG created the H163A mutant enzyme, prepared the protein, and performed the NMR experiment. RSP performed the stopped‐flow experiments and wrote the paper.

## Supporting information


**Fig. S1.** Portion of sequence alignments of DGL from various bacteria.
**Fig. S2.** Negative ion mode ESI‐MS of the product of H163A DGL.

## Data Availability

The data that support the findings of this study are available from the corresponding author (plp@uga.edu) upon reasonable request.
