# Supplementary material for: His‐163 is a stereospecific proton donor in the mechanism of d‐glucosaminate‐6‐phosphate ammonia‐lyase
Source: FEBS Lett. 2022 Aug 29;596(18):2441–8. doi: 10.1002/1873-3468.14469 (PMC9529869; doi:10.1002/1873-3468.14469)
Supplement: Supplementary file 1 — Fig. S1. Portion of sequence alignments of DGL from various bacteria. Fig. S2. Negative ion mode ESI‐MS of the product of H163A DGL. [file FEB2-596-2441-s001.pdf]

Supporting information

His-163 is a stereospecific proton donor in the mechanism of D-glucosaminate-6-phosphate ammonia-lyase

Robert S. Phillips*[§†], Kaitlin L. Anderson[‡], and Declan Gresham[|]

[§]Department of Chemistry, University of Georgia, Athens, GA 30602 USA

[†]Department of Biochemistry and Molecular Biology, University of Georgia, Athens, GA 30602 USA

[‡]Department of Genetics, University of Georgia, Athens, GA 30602 USA

[|]Department of Cellular Biology, University of Georgia, Athens, GA 30602 USA

Table of contents

Figure S1. Portion of sequence alignments of DGL from various bacteria 3

Figure S2. Negative ion mode ESI-MS of the product of H163A DGL 4

```
ref|WP_000188463.1| TSAIRLGGGRIVEVGSSNLATRWQLESAINEKTAALLYVKSHHCVQKGMLSIDDFVQVAQ
ref|WP_020845590.1| SSAIRLGGGRIVEVGSSNLAARWQLESAMTKNTAALLYVKSHHCVQKGMLSIDDFVQVAQ
ref|WP_012135805.1| TSAIRLGGGRVVEVGSSNLAARWQLESAITDDTAALLYVKSHHCVQKGMLGIEDFVQVAQ
ref|WP_061275614.1| TSAVRLGGGRVVEVGSSNLAARWQLESAVTENSAALLYVKSHHCVQKGMLSIEDFVQVAQ
ref|WP_016809673.1| TSAIRLGGGRVVEVGSSNLAARWQLESAISTSTAALLYVKSHHCVQKGMLSIEDFVQVAQ
ref|WP_038162664.1| TSAIRLGGGRVVEVGASNLAARWQLERAVSERTAALLFVKSHHCVQKGMLTIDDFVQVAR
ref|WP_054178914.1| TSAIRLGGGRVVEVGASNLAARWQLERAVSERTAALLFVKSHHCVQKGMLTINDFVQVAR
ref|WP_063446277.1| TSAIRLGGGRIVEVGSSNLAVRWQLQRAVNARTAALLYVKSHHCVQKGMLDIDDFVQVAR
ref|WP_062740258.1| VSAIRLGGGRVVEVGSSNLAARWQLERAVSERSAALLYVKSHHCVQKGMLSIEDFAAVAQ
ref|WP_046449854.1| TSAIRLGGGRIVEVGQSNLAARWQLEKAVSERTAALLFVKSHHSVQKGMLTLEDFVAVAK
ref|WP_046458053.1| TSAIRLGGGRIVEVGQSNLAARWQLEKAVSDRTAALLFVKSHHSVQKGMLTLEDFVAVAK
ref|WP_004962170.1| TTAIRLGGGRVVEVGQSNLAARWQLAKAITAQTAALFYVKSHHCVQKGMVALSDFVALAR
ref|WP_018749325.1| TTMIRLGGGVPVEAGQANEAEPFQVEAAIGERTAALLYVKSHHAVQKGMVPLAEMIRIAR
ref|WP_066496308.1| TQMIKIGGGKIIEVGCANKVEIDHIKEAITDNTAVLFYVKSHHAVQKGMVSLDDMIKIGQ
ref|WP_078712728.1| TSMIRLGGGVPVEVGMANSVAIEDVEQAITDKTCALIYIKSHHCIQKGMISIEKMVEIAH
ref|WP_066497445.1| TQMVRLGGGVPIEIGQANKVQDFHIEDAINDKTAALLYVKSHHAVQKGMVPIEKMVEIAH
ref|WP_015311849.1| NQMIMLGGGIPVEVGQANKVEYQHVEKAINDKTTALIYIKSHHTVQKGMLSIEKMIEIAH
ref|WP_091543875.1| QQMIRLGGGIPHEVGQANKVEINHIKEAITEKTAALFYVKSHHTVQKGMVSIEGMIKIAK
ref|WP_009140813.1| PTMIRLGGGVPVEVGTANEVHPEDIEEAITDKTVALLYVKSHHCVQKGMVGIEAMRDIAH
ref|WP_066505812.1| NQMVRLGGGVVKEVGHSNKTEDFHIGNAINENTAAILYIKSHHTVQKGMLSLEKVCEIAS
ref|WP_059032690.1| VQMVSLGGGRPIEVGQANKTESFHIEGAINEKTAALLFIKSHHTVQKGMVSFQAMADIAH
ref|WP_088066760.1| DQMIRLGGGKVIEVGNANKVEVDHIQEAISDKTAAIFYVKSHHAVQKGMVSVEKMIEIAQ
ref|WP_100042937.1| TQMLRLGGGIPVEAGCSNKVERDNIESAINEKTAALLYVKSHHAVQKGMQSIESMLELAR
ref|WP_095305585.1| AQMVRIGGGKPVEVGNANKVEPDHIEQAITEKTAALLYIKSHHAVQKGMQSLETMMKIAK
ref|WP_072414425.1| PTMIRLGGGVPVEVGCANEVVPADIEEAITAKTAALFYVKSHHCVQKGMVDIETMRDIAH
ref|WP_018579727.1| ATMVRLGGGVPIEAGLVNAVTVADMEAEINENTAGMLYVKSHHTVQKGMIALDAMIKLAH
ref|WP_003387712.1| RQMVALGGGQAIEAGHANHVEEAHLRAEIDEQTAALLFVQSHHAVQKGMQSLETMIQLGR
ref|WP_067211387.1| AQMISLGGGQVVEVGQANKVEAQHIEEAINENTAALMYIKSHHAIQKGMQPIETMIEIAK
ref|WP_066829398.1| PTMIRLGGGVPVEVGCANEVVPADIEEAITDKTVALFYVKSHHCVQKGMVDIETMRDIAH
ref|WP_005979316.1| TTMIRLGGGIPVEAGCSNEVKPEHVEEAIDENTIALLYVKSHHAVQKGMLSIEEMSEIAK
ref|WP_007132603.1| ETMVYLGGGKVVEVGYANEGRKEHIEEAINERTAAILYVKSHHCVQKNMISVEEAAEVAR
ref|WP_006304839.1| TQMIRLGGGHPVEVGYANLVAPVNVENAINDRTAALLYVQSHHAVQKGMVSLAEMIEIAH
ref|WP_005949310.1| TTMIRLGGGIPVEAGCSNEVKPEHVEEAIDENTVALLYVKSHHAVQKGMLSIEEMAEIAH
ref|WP_072969481.1| LQMIRLGGGKVVEVGQTNYVEKNHIIDAISEKTVAIFYVKSHHAVQKGMLSLPEIIEIGE
ref|WP_029097569.1| RQMIALGGGKVVEAGHANHVEEAHLREAIGDRTAALLYVKSHHAVQKGMQPLETMIALGR
consensus       121 ......***...*.*  * .    .. .............*** .**.*....... ..
```

Figure S1. Portion of sequence alignments of DGLs from various bacteria. The sequence at the top is that of Salmonella enteria. The conserved residues are colored magenta.

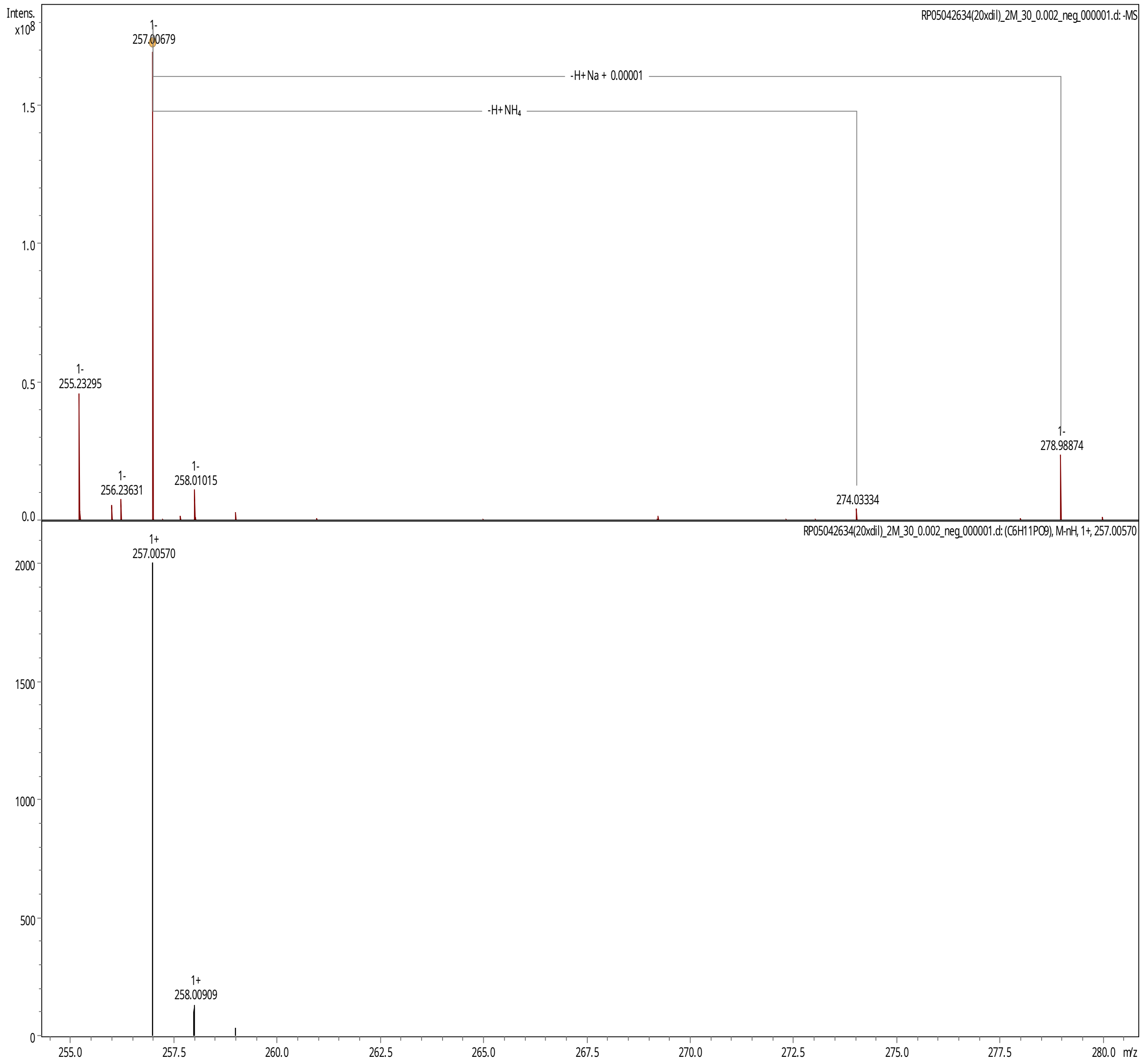

Figure S2. Negative ion mode ESI-MS of the product of H163A DGL.
